# Coordinating With a Robot Partner Affects Neural Processing Related to Action Monitoring

**DOI:** 10.3389/fnbot.2021.686010

**Published:** 2021-08-11

**Authors:** Artur Czeszumski, Anna L. Gert, Ashima Keshava, Ali Ghadirzadeh, Tilman Kalthoff, Benedikt V. Ehinger, Max Tiessen, Mårten Björkman, Danica Kragic, Peter König

**Affiliations:** ^1^Institute of Cognitive Science, Universität Osnabrück, Osnabrück, Germany; ^2^Robotics, Perception and Learning, School of Electrical Engineering and Computer Science, Kungliga Tekniska Högskolan Royal Institute of Technology, Stockholm, Sweden; ^3^Donders Institute for Brain, Cognition and Behaviour, Radboud University, Nijmegen, Netherlands; ^4^Stuttgart Center for Simulation Science, University of Stuttgart, Stuttgart, Germany; ^5^Institut für Neurophysiologie und Pathophysiologie, Universitätsklinikum Hamburg-Eppendorf, Hamburg, Germany

**Keywords:** human-robot interaction, social neuroscience, joint action, ERP, EEG, embodied cognition, action monitoring

## Abstract

Robots start to play a role in our social landscape, and they are progressively becoming responsive, both physically and socially. It begs the question of how humans react to and interact with robots in a coordinated manner and what the neural underpinnings of such behavior are. This exploratory study aims to understand the differences in human-human and human-robot interactions at a behavioral level and from a neurophysiological perspective. For this purpose, we adapted a collaborative dynamical paradigm from the literature. We asked 12 participants to hold two corners of a tablet while collaboratively guiding a ball around a circular track either with another participant or a robot. In irregular intervals, the ball was perturbed outward creating an artificial error in the behavior, which required corrective measures to return to the circular track again. Concurrently, we recorded electroencephalography (EEG). In the behavioral data, we found an increased velocity and positional error of the ball from the track in the human-human condition vs. human-robot condition. For the EEG data, we computed event-related potentials. We found a significant difference between human and robot partners driven by significant clusters at fronto-central electrodes. The amplitudes were stronger with a robot partner, suggesting a different neural processing. All in all, our exploratory study suggests that coordinating with robots affects action monitoring related processing. In the investigated paradigm, human participants treat errors during human-robot interaction differently from those made during interactions with other humans. These results can improve communication between humans and robot with the use of neural activity in real-time.

## 1. Introduction

We constantly interact with other humans, animals, and machines in our daily lives. Many everyday activities involve more than one actor at once, and groups of interacting co-actors have different size. Especially, interactions between two humans (so-called dyadic interactions) are the most prevalent in social settings (Peperkoorn et al., 2020). During such situations, we spend most of our time trying to coordinate our behavior and actions with other humans. Until recently, human cognition was mostly studied in non-interactive and single participant conditions. However, due to novel conceptual and empirical developments, we are now able to bring dyads instead of single participants to our labs (Schilbach et al., 2013). This approach is called Second-person neuroscience (Schilbach et al., 2013; Redcay and Schilbach, 2019). It suggests that we need to study the social aspect of our cognition with paradigms that include real-time interactions between participants instead of the passive observation of socially relevant stimuli (Redcay and Schilbach, 2019). Such an approach can reveal a new perspective on human social cognition.

Coordination between members of a dyad is achieved by joint actions (Sebanz and Knoblich, 2021). There are different aspects of coordination that facilitate achieving common goals between co-actors. Firstly, Loehr et al. (2013) showed in pairs of pianists performing solo and duets that monitoring of our actions, our partner's actions, and our joint actions is required to coordinate successfully. Second, being familiar with each co-actors individual contributions in the dyad helps to form predictions about the partner's actions, which further improves coordination (Wolf et al., 2018). Third, recently proposed action-based communication serves as a fundamental block of coordination (Pezzulo et al., 2013). In comparison to verbal communication, this low-level sensorimotor communication is implicit and faster. Experiments by Vesper et al. (2017) serve as examples of sensorimotor communication in the temporal dimension. Their results have shown that participants adjusted their actions to communicate task-relevant information. Fourth, while both co-actors are engaged in a constant flow of perceptual information, they create coupled predictions about each other's actions that are necessary to achieve fruitful coordination (Sebanz and Knoblich, 2021). Curioni et al. (2019b) investigated coordination tasks with incongruent demands between partners, and their results suggested the benefits of reciprocal information flow between participants. In sum, there are different aspects of human cognition that allow for the maintenance of dyadic coordination: Action monitoring, predictions based on familiarity of partner's actions, action-based communication, and reciprocal information flow.

So far, most dyadic interaction studies investigated the coordination between human co-actors (Sebanz et al., 2006; Vesper et al., 2010). However, in recent years we are more and more surrounded by robotic co-actors (Ben-Ari and Mondada, 2018). Furthermore, there are many different predictions for the future of robotics, but all point into the same direction: there will be more robots among us (Stone et al., 2016; Diamond, 2020; Wiederhold, 2021). In line with this, humanoid robots are getting progressively better at socially relevant tasks (Campa, 2016). It is thought that these social robots will be used in many different fields of our everyday life in the upcoming years (Enz et al., 2011). One of the main challenges in robotics is creating robots that can dynamically interact with humans and read human emotions (Yang et al., 2018). Concerning these changes in our environment, a new research line has emerged and already substantially contributed to our understanding of human-robot interactions (Sheridan, 2016). As many different scientists are slowly approaching this topic, the field of human-robot interaction until now focused on human thoughts, feelings, and behavior toward the robots (Broadbent, 2017). Studying these specific aspects is essential and further, we believe that the scientific community has to investigate real-life interactions between humans and robots in order to fully understand the dynamics that underlie this field. Therefore, we propose to use both human and robot partners in experimental paradigms as this will help to close the gap in understanding dyadic interactions.

There are different tools and methods to study the social brain and behavior (Krakauer et al., 2017): EEG (Luck and Hillyard, 1994), fMRI (Eisenberger, 2003), MEG (Baillet, 2017), and fNIRS (Ferrari and Quaresima, 2012). From this list, Electroencephalography (EEG) stands out as particularly useful for studying dynamical interactions, as it not only aligns with the temporal resolution of social interactions, but also allows for free movement and thereby allows for dynamic interactions. This temporal resolution allows studying brain processes with milliseconds precision. One of the methods that are classically used within EEG research are event-related potentials (ERPs) (Luck and Hillyard, 1994). ERPs are suitable to study different components of brain processes while they evolve over time. The classic study by Miltner et al. (1997) showed different brain signatures for correctly and incorrectly performed trials at around 200-300 milliseconds after the feedback about an action was perceived. This brain component was named Feedback related negativity (FRN). In similar studies, van Schie et al. (2004) showed that the FRN is sensitive not only to our own actions but also those of others. Czeszumski et al. (2019) further extended this finding to different social contexts (cooperation and competition). Thus, EEG and specifically ERPs have been proven valuable tools to investigate the physiological basis of social interactions.

Therefore, we have a good understanding of EEG-based markers of action monitoring. Nonetheless, it is only in recent years that human behavior and its neural basis are studied together with robotic partners (Wykowska et al., 2016; Cheng et al., 2020). Based on more than 20 years of research on action monitoring in humans, similar ERP components (E/FRN) were expected to be elicited in human-robot paradigms. Namely, the difference between brain responses to correct and incorrect actions of a robotic arm was found (Iturrate et al., 2015; Kim et al., 2017). Furthermore, these differences in midfrontal ERP components were used to improve co-adaptation between human and robot behavior in turn-taking tasks (Salazar-Gomez et al., 2017; Ehrlich and Cheng, 2018, 2019a,b; Iwane et al., 2019), and real-world driving (Zhang et al., 2015; Chavarriaga et al., 2018). Such EEG based interfaces highlight the importance of studying the neural basis of human-robot interactions. The results confirm that similar brain mechanisms are involved when we observe actions of the robot. Yet, little is known about action monitoring in dynamic situations with non-human, robotic partners. The goal of this study was to test whether the same neural mechanisms are present when we interact with robots in a dynamic paradigm and if there are differences between human and robotic partners.

To answer these questions, we adapted a dynamic dyadic interaction paradigm for human-robot interactions. We chose the paradigm from Hwang et al. (2018) and Trendafilov et al. (2020), in which two human participants had to manipulate a virtual ball on a circular elliptic target displayed on a tablet and received audio feedback of the ball's movement. Participants used their fingers to move the tablet and manipulate the position of the ball. We changed the paradigm, by adapting the tablet to enable coordination with the robot and to fit the requirements for EEG measurements. On the one hand, this paradigm allows for coordination similar to a real-life situation; on the other hand, it allows for the analysis of neural underpinnings of cognitive functions required for coordination. In this study, we specifically focused on the aspect of action monitoring with human and robot partners. Thus, to extend our knowledge the present study investigates action monitoring in a dynamic interaction task between humans and robots. Additionally, based on the results from Hwang et al. (2018) we decided to test whether auditory feedback about actions (sonification) influences coordinated behavior and cognitive processes. Taken together, this study tries to approach a novel problem with interdisciplinary methods and sheds new light on the neural processes involved in dynamic human-robot interactions.

## 2. Methods

### 2.1. Participants

We recruited 16 participants (7 female, mean age = 25.31±1.92 years) from KTH Stockholm Royal Institute of Technology. We had to exclude two dyads from further analysis, one due to measurement errors in the robot control and one due to excessive movements from participants which led to large artifacts in the EEG data, leaving data from 12 participants in 6 recording sessions. Participants had normal or corrected-to-normal vision and no history of neurological or psychological impairments. They received course credits for their participation in the study. Before each experimental session, subjects gave their informed consent in writing. Once we obtained their informed consent, we briefed them on the experimental setup and task. All instructions and questionnaires were administered to the participants in English. The Swedish Ethical Review Authority (Etikprövningsnämnden) approved the study.

### 2.2. Task and Apparatus

**Figure 1:**
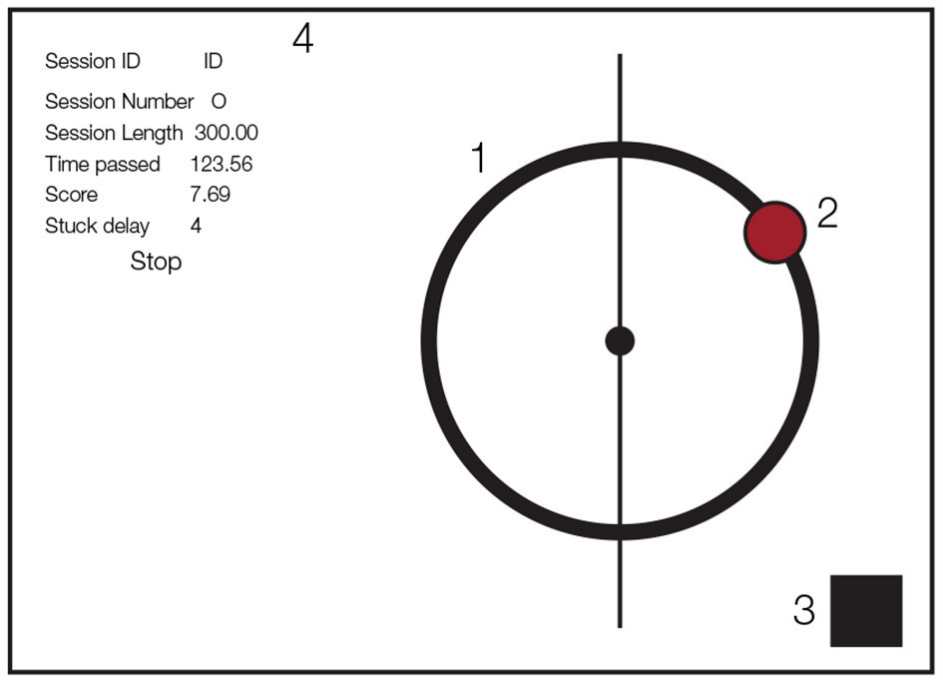
Schematic of game design on the tablet. (1) Circular track, (2) ball, (3) flashing rectangle indicating experimental events (covered by luminance sensor), (4) text box for experiment monitoring (only used by experimenter).

During each recording session, participants performed the task in four blocks of 10 min each, twice with a human partner and twice with the robot. Further, each dyad (partner human or robot) performed the task with or without auditory feedback (sonification on or sonification off). The task was based on a tablet game where the dyads cooperated with each other to balance a ball on a circular track as they simultaneously moved it in counter-clockwise direction (Hwang et al., 2018) (Figure 1). At random intervals, we added perturbations that radially dispersed the position of the ball away from the current position. In order to reduce the subjects' expectations of the occurrence of the perturbations, we sampled its rate of occurrence from a Poisson distribution with λ = 4 s.

The experimental task was implemented on an Apple IPad Air tablet (v2, 2048 × 1536 pixel resolution, refresh rate 60 Hz) using Objective-C for iOS. During the task, subjects saw a red ball of 76.8 pixel radius on a circular track with a radius of 256 pixels and a thickness of 42.67 pixels. The ball position was represented as the horizontal and vertical coordinates with respect to the center of the circular track (0,0). The tablet was mounted on a metal frame of size 540 × 900 mm. We further added a square of size 100 × 100 pixels that was used as a signal source for, and covered by, a luminance sensor.The luminance sensor is a light-sensitive diode that converts light into electrical current. We changed the color of a small patch on the tablet for the different events in the experiment (start of the experiment, start of a perturbation, end of the experiment) over which the luminance sensor was placed. Figure 1 shows all the visual components displayed to the participants (the text box on the left side was used by the experimenter to monitor the experiment status).

**Figure 2:**
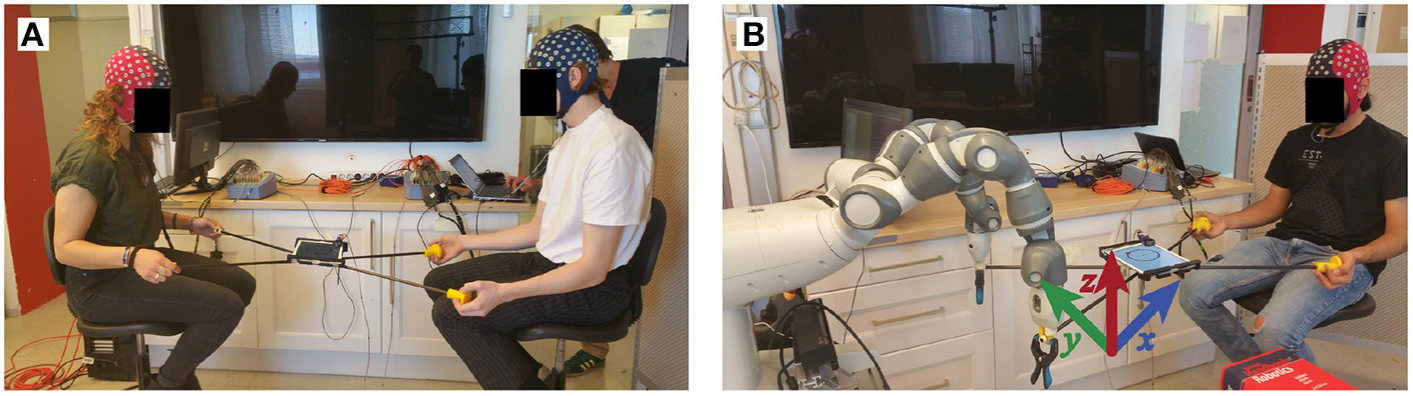
Experimental setup. Participants performed the experiment with another participant **(A)** or a robot partner **(B)**. In each condition they played a tablet game by balancing a virtual ball on a circular track while moving it in the counter-clockwise direction.

During the periods with another human partner, we asked the participants to not verbally interact with each other. During the task, they sat face-to-face at 1m distance as they held handles connected to the short end of the frame. Similarly, while performing the task with the robot, subjects held the short end of frame while the other end of the frame was clamped to the grip effectors of the robot. Figure 2 shows the physical setup of the subjects and the robot during the experiment.

For the periods involving sonification, the position and angular velocity of the ball were sonified. The auditory feedback was created by a Gaussian noise generator with a band-pass filter (cut-off frequency: ±25 Hz). The horizontal and vertical coordinates of the ball modulated the pitch of the auditory feedback, while its angular velocity modulated the loudness. The sonification procedure was implemented using the specifications provided in Hwang et al. (2018).

Lastly, we used a self-manufactured luminance sensor that synchronized the experimental events (experiment start and end, and perturbation) between the tablet and the EEG amplifier. We changed the luminance source color from black to white to mark the start of the trials, white to black to mark the end of the trials. During a session the patch was white, except at the frame where the perturbation happened, which was marked with gray (RGB = 134, 134, 134).

### 2.3. Robot Control

We used the YuMi robot (ABB, Västerås, Sweden) shown in Figure 2 for our experiments. We implemented a Cartesian space controller based on the original joint-level velocity controllers provided by the manufacturer. The robot had direct access to the tablet data and no active sensing was necessary. Starting the robot at the joint position depicted in the Figure 2, we send Cartesian space velocity commands to both arms at 10 Hz. The Cartesian controller was designed such that the *X, Y* positions of both end-effectors are kept constant during an execution, and only the *Z* position of the end-effectors are adjusted to move the ball. We denote the left and right end-effector velocity commands in the *z* axis by $v_{l}^{z}$ and $v_{r}^{z}$ and the current *X, Y* position of the ball on the game by (*b*_*x*_, *b*_*y*_), respectively. We first obtain the angle θ corresponding to the current position of the ball in the polar coordinate system by θ = *arctan*(*b*_*y*_, *b*_*x*_). Then, we obtain the next target angle $\hat{\theta }=\theta +\pi /12$ to let the ball move in the counterclockwise direction. The next target *X,Y* positions of the ball are found as ${\hat{b}}_{x}=G_{p}\left(R\times cos\left(\hat{\theta }\right)-b_{x}\right),{\hat{b}}_{y}=G_{p}\left(R\times sin\left(\hat{\theta }\right)-b_{y}\right)$, where *R* is the radius of the circle on the IPad game and *G*_*p*_ = 0.1 is a constant gain. The velocity commands in the *z* axis are then found as $v_{l}^{z}=-G_{v}\left({\hat{b}}_{x}-\alpha_{x}\right)-G_{v}\left({\hat{b}}_{y}-\alpha_{y}\right),v_{r}^{z}=G_{v}\left({\hat{b}}_{x}-\alpha_{x}\right)-G_{v}\left({\hat{b}}_{y}-\alpha_{y}\right)$, where, α_*x*_, α_*y*_ are gravity acceleration in the *X, Y* directions measured by the IPad, and *G*_*v*_ = 0.5 is a constant gain. The command velocities are then clipped to have an absolute value less than 0.02 m/s, and the clipped values are sent to the Cartesian velocity controller.

### 2.4. Experimental Protocol

We prepared both participants for the EEG recording together, which took around 45 min to complete. Once the subjects were ready to start the experiment, we led them to a room that housed the robot. Depending on the dyad combination, we provided oral instructions about the task and clarified any remaining questions. For human-human dyads, we started the task on the tablet with either of the sonification conditions depending on the experiment session. To counterbalance the sonification and partner sequence for the combinations of dyads (human-human or human-robot), we permuted the combinations. Each experimental session was sequenced based on this permutation. We also counter-balanced the sonification during the task, so that every even numbered experiment session started with the sonification condition for all the dyad combinations. For the human-robot dyads, we first reset the limbs of the robot to its initial conditions and then started the task on the tablet. After each block, the participants were given a short break and then repeated the task with the alternate sonification condition. The whole experimental session lasted for about 4 h.

### 2.5. EEG Data Acquisition

We recorded the EEG using two 64-Ag/AgCl electrode systems (ANT Neuro, Enschede, Netherlands), and two REFA8 amplifiers (TMSi, Enschede, Netherlands) at a sampling rate of 1,024 Hz. The EEG cap consists of 64 electrodes placed according to the extended international 10/20 system (Waveguard, eemagine, Berlin, Germany). We placed the ground electrode on the collar-bone. We manually adjusted the impedance of each electrode to be below 10kΩ before each session. The recording reference was the average reference, which, only in the single-brain recordings, was later programatically re-referenced to Cz. During human-human interactions, two brains were recorded simultaneously with the separate amplifiers, synchronized through the ANT-link (Synfi, TMSi, Enschede, Netherlands). VEOGs were recorded with two additional electrodes, one placed below and one above the eye.

### 2.6. Pre-processing

The analysis of the EEG data was performed in MATLAB 2016b and the behavioral analyses in Python 3.7.

We preprocessed the data using the EEGLAB toolbox (v2019.0) (Delorme and Makeig, 2004). As a first step before preprocessing, we programmatically extracted the trigger events from the luminance sensor and added them to the recorded data. Then, the data from each condition was downsampled to 512Hz, followed by referencing all datasets to Cz electrode. We then high-pass filtered the dataset at 0.1Hz and then low-pass filtered it at 120 Hz in order to not unnecessarily discard gamma frequency activity (6 dB cutoff at 0.5 Hz, 1 Hz transition bandwidth, FIRFILT, EEGLAB plugin, Widmann et al., 2015). Following this, we manually removed channels that showed strong drift behavior or excessive noise (mean: 7, SD: 2.7, range: 1–13). We manually inspected the continuous data stream and rejected the portions which exhibited strong muscle artifacts or jumps. To remove further noise from eye and muscle movements, we used independent component analysis (ICA) based on the AMICA algorithm (Palmer et al., 2008). Before performing ICA, we applied a high-pass filter to the data at 2Hz cut-off to improve the ICA decomposition (Dimigen, 2020). We visually inspected the resulting components in combination with using ICLabel (Pion-Tonachini et al., 2019) classifier. IClabel was run on epoched data, 200 ms before and 500 ms after the perturbation. Based on the categorization provided by ICLabel, and a visual inspection of the time course, spectra, and topography, we marked ICs corresponding to eye, heart and muscle movements for rejection (mean: 26.5, SD: 5.2, range: 18–44). We copied the ICA decomposition weights to the cleaned, continuous data and rejected the artifactual components. Finally, using spherical interpolation, we interpolated the missing channels based on activity recorded from the neighboring channels.

### 2.7. Behavioral Analysis

To understand the behavioral differences for the factors partner and sonification, we used measures of mean angular velocity and mean error produced. These behavioral differences indicate how well the partners coordinated with each other. Furthermore, as the velocity and position of the ball were sonified, these measures are indicative of the effect of sonification on the dyadic performance. We first calculated the instantaneous angular position θ (in degrees) of the ball using the horizontal and vertical (X, Y) positions of the ball on the tablet as follows:

(1) $$\begin{array}{l} \theta_{t}=\frac{180}{\pi }*\arctan\frac{y_{t}}{x_{t}} \end{array}$$

We used the atan2 function to take into account the X, Y position in the negative coordinate axes. θ_*t*_ values were transformed from [−π, π] to range [0, 2π]. Next, we computed the instantaneous angular velocity ω of the ball using the following formula where *t* is the sample time-point:

(2) $$\begin{array}{l} \omega =\frac{\Delta \theta }{\Delta t} \end{array}$$

We, subsequently, calculated the mean ω for each participant for the four different conditions. Next, We calculated the error as the difference of the instantaneous radial distance between the radius of the track and the ball's current position measured as the distance from the track's center as follows:

(3) $$\begin{array}{l} error_{t}=\sqrt{x_{t}^{2}+y_{t}^{2}}-Radius_{track} \end{array}$$

### 2.8. Deconvolution and EEG Analysis

Even though the perturbations were sampled from a Poisson distribution with λ = 4, the corresponding neural responses might overlap in time and bias the evoked potentials (Ehinger and Dimigen, 2019; Dimigen and Ehinger, 2021). Further, experimental block onset and offset typically elicit very strong ERPs overlapping with the perturbations. Finally, we see clear, systematic differences in the behavior depending on the condition (e.g., higher velocity with a human partner), which could lead to spurious effects in the ERPs. We further added eccentricity (distance from the circles midpoint), in order to control for the ball's trajectory. In order to control both temporal overlap and covariate confounds, we used linear deconvolution based on time-regression as implemented in the unfold toolbox v1.0 (Ehinger and Dimigen, 2019). Consequently, we modeled the effects of the partner (human or robot), the sonification (off = 0, on = 1) and their interaction as binary, categorical variables, the eccentricity and the velocity were coded using B-spline basis functions and the angular position using a set of circular B-splines. The block on- and offsets were modeled as intercept only models. The complete model can be described by the Wilkinson notation below (Wilkinson and Rogers, 1973).

$$\begin{array}{lll} perturbation\;ERP & \sim & 1+partner+sonification+partner:sonification\\ +\;circularspline\left(angular\;position,8\right)\\ +spline\left(eccentricity,5\right)\\ +\;spline\left(velocity,5\right)\\ block\;onset\;ERP & \sim & 1\\ block\;offset\;ERP & \sim & 1 \end{array}$$

This model was applied on the average referenced continuous EEG data, and each event was modeled in the time range of −500 to 700 ms with respect to the event onset. We collected a mean value of 640 trials per subject.

Similar to the two-stage mass univariate approach, we calculated the t-value over subjects for each of the resulting regression coefficients (similar to difference waves between two conditions) for all electrodes and time points (time-range of −500 to 700 ms). That is, for the purpose of comparison of two conditions, they are preferable as they avoid confounds by other factors. The multiple comparison problem was corrected using a permutation based test with threshold-free cluster enhancement (TFCE) (Mensen and Khatami, 2013; Ehinger et al., 2015) with 10,000 permutations(default parameters *E* = 0.5 and *H* = 2). We used the eegvis toolbox (Ehinger, 2018) to visualize all evoked response potentials.

## 3. Results

### 3.1. Behavioral

**Figure 3:**
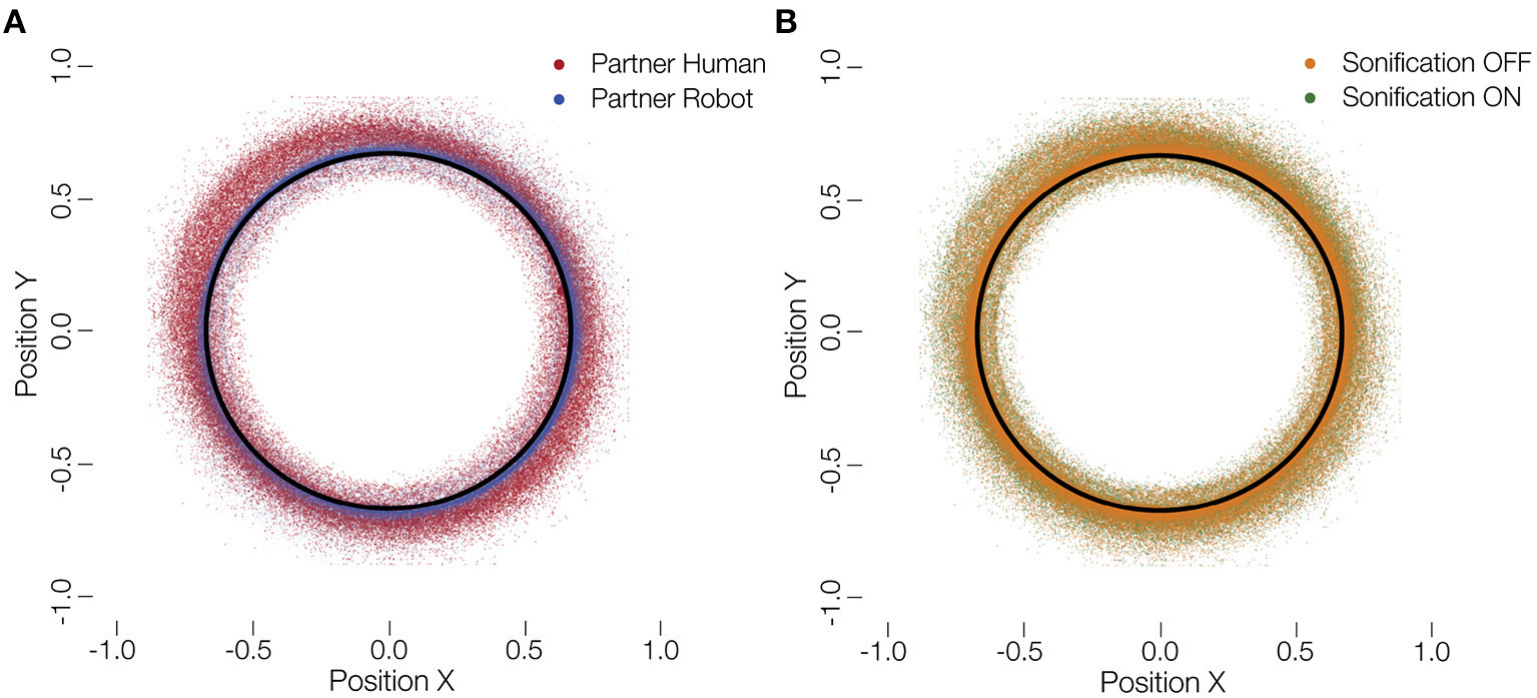
Distribution of ball positions. **(A)** Ball positions on the tablet with a human (red) and robot (blue) partner. **(B)** Ball positions on the tablet for sonified (green) and not sonified movements (orange). The black circle represents optimal trajectory. It can be seen that participants deviated more with a human partner. No such difference is visible for a change in the sonification.

In this study, humans played a collaborative game either with other humans or with robots. We further added sonification of the ball's movement as a supplementary auditory feedback to the participants. Figure 3 shows the raw positions of the ball overlaid for all subjects and the partner and sonification conditions. The behavior we analyse here, is the mean velocity of the ball during each session and the mean deviation of the ball from the circular track. These measures indicate how fast the participants performed the task and how much error they produced, both a proxy of the success of the collaboration.

**Figure 4:**
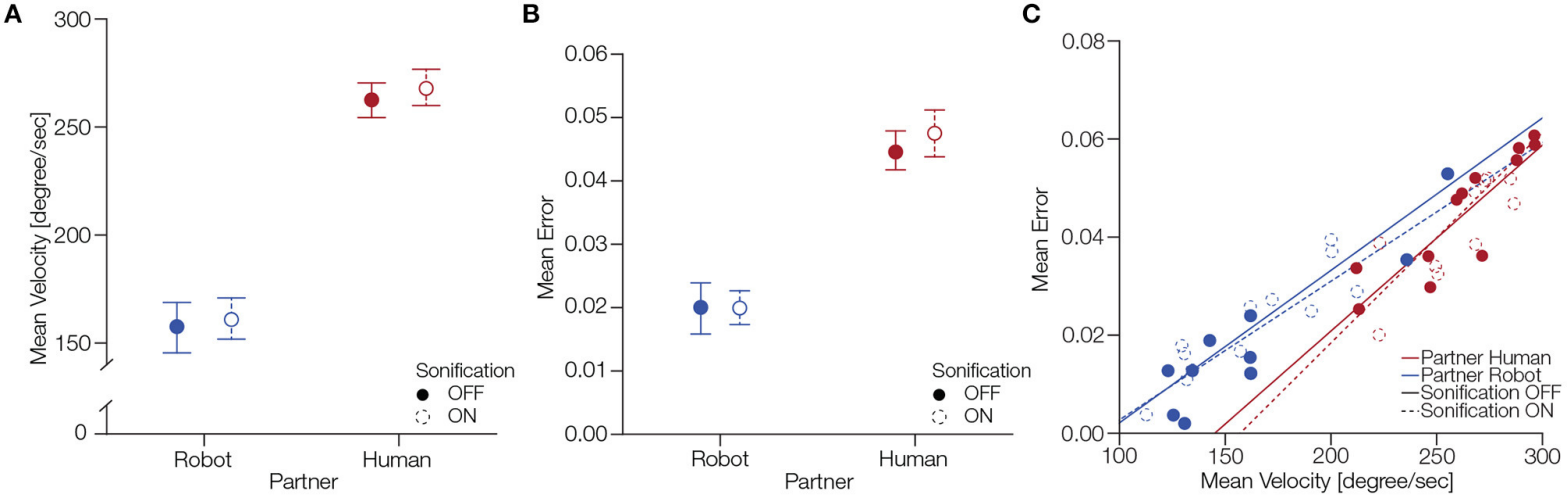
Behavioral differences between conditions partner and sonification. **(A)** Differences in mean angular velocity across different participants. The error bars indicate standard error of mean. **(B)** Differences in mean error across different participants. The error bars indicate standard error of mean. Panel **(C)** shows the correlation of mean error and mean velocity for partner and sonification conditions.

We calculated the mean angular velocity (ω) for each participant for the four different conditions (Figure 4A). To test the statistical significance of these findings, we computed a 2 × 2 factorial repeated measures ANOVA with the factors partner and sonification. The ANOVA showed a significant main effect of partner, *F*_(1, 11)_ = 87.09, *p* < .0001 where subjects exhibited a mean angular velocity of 265.20 degrees/second and SD ±0.28.29 with a human partner, conversely, with a robot partner subjects showed a mean angular velocity of 159.23 degrees/second ±29.40. The ANOVA did not reveal a significant main effect of sonification, *F*_(1, 11)_ = 1.00, *p* = 0.33, with mean angular velocity 210.06 degrees/second ±65.51 with sonification off and the mean angular velocity was 214.36 degrees/second ±62.53 with sonification on. There was no significant interaction of factors partner and sonification, *F*_(1, 11)_ = 0.04, *p* = 0.83. Hence, we can conclude that participants were faster at moving the ball on the circular track while performing the task with a human partner.

Next, we analyzed the mean error produced by participants during a session. Figure 4B shows the mean error across participants for the four different conditions. To statistically assess these differences, we performed a 2 × 2 factorial repeated measures ANOVA with factors partner and sonification. The ANOVA revealed significant main effect for partner *F*_(1, 11)_ = 42.61, *p* < 0.0001 where subjects had a mean error of 0.04±*SD* = 0.012 while performing with a human partner, conversely, they had a mean error of 0.01±0.012 while cooperating with the robot. We did not find a significant main effect of sonification *F*_(1, 11)_ = 1.75, *p* = 0.21 where subjects had a mean error of 0.032±0.017 with the sonification off and mean error of 0.033±0.018 with sonification on. There was no significant interaction of factors partner and sonification, *F*_(1, 11)_ = 0.51, *p* = 0.48. We can conclude that subjects made larger errors while performing the task with a human partner compared to the robot partner.

Lastly, we were interested in the correlation between the behavioral measures we analyzed. Figure 4C shows the correlation of mean error and mean velocity for the partner and sonification conditions. For human partner with sonification off the Pearson correlation showed a correlation coefficient ρ = 0.98, *p* < 0.001 and for sonification on ρ = 0.89, *p* < 0.001. For robot partner with sonification off ρ = 0.97, *p* < 0.001 and with sonification on ρ = 0.97, *p* < 0.001. These results show that the mean error and mean velocity were positively correlated during the task.

### 3.2. EEG

**Figure 5:**
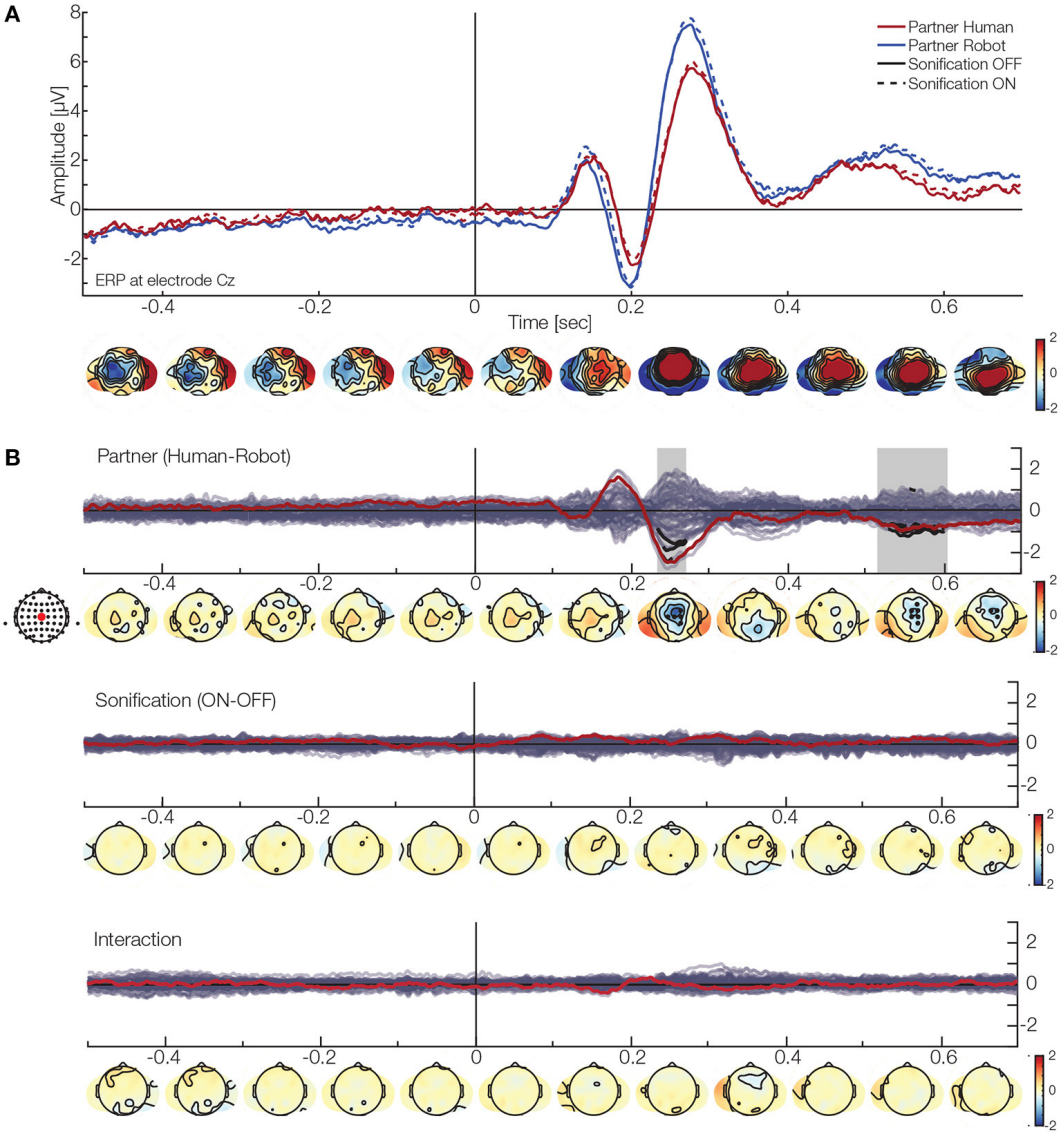
EEG results. **(A)** ERP at electrode Cz. The red lines show the activation when interacting with a human partner, while blue lines indicate a robot partner. The solid line are the ERP when the sonification was off, while the dashed line represent sonification on. Below, are the topographies for the grand average (mean over all conditions). **(B)** Clustering results for the different factors (red line and dot represents electrode Cz). Top: Effect of partner. The analysis finds two clusters in the central area (black dots and segments). One is likely due to a difference at around 230–270 ms, while the second one is present later (around 510–600 ms). These results indicate that the ERP will have a smaller amplitude when interacting with a human partner. Middle: Effect of sonification. No cluster was found here. Bottom: Interaction. No cluster was found here.

Next, we look at the overlap- and behavior-corrected brain activity during the task. Using a overlap-corrected time regression approach, we investigate the main effect and interaction ERPs from the 2 × 2 design, while adjusting for eccentricity, velocity and position of the ball (see section 2 for details). For the effect of the behavioral data on the ERP, please see the Supplementary Material. We only report ERPs time-locked to perturbation events. Descriptively, in electrode Cz (Figure 5A), we see the typical pattern of a positive deflection, followed by a negative and a second positive deflection after the perturbation onset. We did not have a specific hypothesis to a predefined component and analyzed all electrodes and time points simultaneously. The TFCE analysis reveals two clusters for the main effect of the factor of partner (Figure 5B). The first cluster is likely to represent the activity between 230 and 270 ms with its maximum amplitude being −2.8μV at electrode FC1 (median p: 0.025, minimal *p*: 0.018). The second cluster most likely represents the time range of 515–605 ms with a peak at −1.2μV at electrode FC2 (median *p*: 0.026, minimal *p*: 0.002). Both clusters are found in the central region. No significant clusters were found for neither the factor sonification nor the interaction term.

These results show that we find differences in the participants' ERPs with respect to their current partner independently of their differences in behavior: When interacting with a robot partner the ERP will have a stronger amplitude indicating a systematically different processing.

## 4. Discussion

Our experiment investigated neural correlates of action monitoring in a dynamic collaboration task that involves two co-actors. Participants performed the task with another human and robot partner while we measured EEG signals. Co-actors tried to keep a virtual ball on the circle displayed on a tablet; they used their hands (human arm or robotic arm) to manipulate independent orientation axes of the tablet. We perturbed the ball to investigate neural action monitoring processes of the participants. We found fronto-central ERP components at around 200–300 ms after the ball was perturbed. The components were stronger for human and robot partner compared to interactions with another human. These results suggest that the dynamic processing of our actions is influenced by whether we collaborate with a robot or a human.

The behavioral measures of our participants' actions were different between human and robot partners. We focused our analysis on two aspects of collaboration: The speed which is represented by the ball's velocity and the accuracy as indicated by the mean error. Our results suggest that participants perform slower when paired with the robot and achieve higher accuracy (ball closer to the circular track). There is a trade-off relation between these factors; this is why we discuss them together (Figure 4C). One simple explanation could be that the robot's control were themselves slow and prone to error. The human participants might have restrained themselves and thereby executed artificially slow movements. Another interpretation of why our participants slow down (and increased accuracy) while performing with the robot is that they had less trust in the robot than a human partner. This is in line with past research that suggests that level of trust changes during real-time interactions with robots (Desai et al., 2013) and that, in general, trust levels are different for human and robot partners (Lewis et al., 2018). Another interpretation for slower movements is that it is challenging to create a model of a partner's actions during a joint collaborative task with a robot. Based on work suggesting that we represent others' actions as our own (Sebanz et al., 2003), it is possible that in the case of interacting with a robot we need more time to create such representations. There is much space for interpretations why having a robot partner triggered slower movements; however, we would like to point that the main goal of our study was to investigate neural correlates of different partners, and behavioral responses were collected to exclude their influence on neural responses (see section 2.8 for details).

After adjusting for behavioral differences in the EEG analysis, we see that robot partners affect neural correlates of action monitoring differently in comparison to a human partner. We found that between 200-300 ms after the perturbation event disturbing the collaboration, the EEG amplitudes differ at the fronto-central sites. The literature on single participants at these electrodes and time window suggests that it is when and where monitoring our errors or feedback about our actions unravels (Miltner et al., 1997; Cavanagh et al., 2009). Similarly, when it comes to neural activity involved in action monitoring in dyadic situations, the same activations play a role (van Schie et al., 2004; Czeszumski et al., 2019). If the error is committed by the participant and can be inferred from his action (e.g., making a typo), the brain component involved is called Error-related negativity, with more negative activation for erroneous actions than correct ones (Yeung et al., 2004). In case of behavior that needs feedback to understand the consequences of the action (for example, gambling task), it is called Feedback related negativity (Hajcak et al., 2006). In comparison to these classic, static, and passive experiments, we had real-time collaboration between two participants, and we observed similar component peaking around 200-300 ms after the perturbation happened. Our participants were not informed about the perturbations, so they could have been treated as participants' own or the partner's error. Therefore, we suggest that the neural activation we observe in our study resembles classic components. Our finding that robotic partners modulate action monitoring corroborates recent study (Hinz et al., 2021). However, there is a crucial difference between both studies: Participants in Hinz et al. (2021) study performed a task sequentially (turn-taking), while in our study, participants interacted with each other in real-life. Both studies point in the same direction. Robot partners modulate neural activity. We speculate that differences in the amplitudes of the ERP for robotic and human partners may arise from differences in how we represent actions of artificial and human-like agents. Such differences might involve partly non-overlapping neuronal substrates with different visibility to EEG recordings. Furthermore, the perceived options to optimize performance in the joint interaction by adjusting to the behavior of the partner might differ. Such differences can elicit different neural patterns that we are able to measure with EEG.

Our results suggest that robot partners can modulate neural activity in a dyadic experiment. Concerning that there is not many studies that focused on neural underpinnings of human-robot interactions, the results we present here have a value for research topics in the field of join-action. They are a first exploratory step toward a theoretical and methodological foundation. We showed the feasibility of conducting a human-robot interaction study while measuring EEG from the human participant in a dynamical paradigm. With full experimental control, we explored neural correlations of human-robot interactions in an ecologically valid setup (Matusz et al., 2019; Czeszumski et al., 2020; Nastase et al., 2020). There is vast literature on the topic of joint actions between humans and robot partners (Curioni et al., 2019a; Schellen et al., 2021; Wahn and Kingstone, 2021). Neural markers of action monitoring during human-robot interactions were studied in turn-taking tasks and utilized for brain-computer interfaces to improve communication between robots and humans (Ehrlich and Cheng, 2018, 2019a,b). Our study shows that it is possible to conduct studies with non-human agents collaborating with humans in real-time and measure brain activity and that the neural basis of action monitoring is affected by the robot partner.

Lastly, we observed small differences between human and robot partners at later time points (between 500-600 ms after the perturbation) around the midline electrodes. These differences are difficult to interpret. The topography suggest similar source as the component discussed above. However, based on time we speculate it could be P3b component. Huberth et al. (2019) reported similar component in study that investigated self and other (human vs. computer) generated actions in pianists. They found that P3b component was present only for self generated actions, suggesting greater monitoring of self generated actions. It is important to highlight that in our study, participant had to dynamically perform the task, while in the Huberth et al. (2019) study participants took turns to perform joint actions. What is similar is that they had to generate actions to achieve a common joint goal (Vesper et al., 2010). It is possible that the late effect we found in our experiment has the same function (greater monitoring of self generated actions). However, in comparison to the earlier effect (200–300 ms after the perturbation), the size of the effect in our study is small. Therefore, we have to be careful with interpretations. Future researcher with bigger sample size can help to understand the function of late ERP components in joint actions with robots.

### 4.1. Limitations

The exploratory aspect of investigating neural underpinnings of human-robot interactions pose many challenges and questions. In the present study, we tried our best to reconcile all of them. However, there are limitations that have to be addressed. First, our sample size was small in terms of number of dyads. However, it was not small in terms of recordings and total amount of gathered data. Thus, the effects reported are significant. Second we did not perform statistic al analyses with a predefined hypothesis. Instead, we performed an exploratory analysis that encompasses all electrodes and time points. It is important to understand that it is the first study of its kind. Therefore, it has to be replicated and evaluated by future research (Pavlov et al., 2021). Third our results could be dependent on the robot used in the study. We suggest that different types of robots (less/more humanoid) could modulate action monitoring differently. The robot used in the present study was clearly not-humanoid. Participants could clearly recognize it as a robot and devoid of typical human traits that are often used in communication/collaboration. Nonetheless, using this robot helped us to maximize the difference between conditions. Additionally, our claim is supported by research on a different level of trust depending on the appearance of humanoid robots (Haring et al., 2013; van Pinxteren et al., 2019). Therefore, it would interesting to perform a similar experiment and compare the results with a more human-like robot. Fourth, as discussed below, our robot did not have a model of the human actor. By this, the robot's behavior helped to boost the characteristic differences between the player's partners. Fifth, our statistical analysis does not take the dyadic dependency into account, possibly biasing the estimated model parameters of the human-human condition downward. In the future, study with a bigger sample size, could answer the question whether dyadic dependencies play a role in the effects reported in our study. Sixth, even though participants were asked to keep their eyes on the center of the circular track, we did not control for eye-movements in this study, which could result in biased viewing-behavior on the tablet. However, we adjusted for ball position while modeling the ERPs, which is likely to be a proxy for current eye position and also remove eye movement and blink related ICs. Furthermore, the game required constant attention and engagement, so it was assured that participants did not look away from the tablet and the ball. Additionally, we are interested in the EEG signal related to the behavior, rather than the visual stimulus. All in all, we addressed the limitations, and are convinced that they do not impede the interpretations of our results as presented in next paragraphs.

## 5. Conclusions

Taken together, this study explored and described event-related potentials related to action monitoring in humans collaborating with other humans or robots. We used a dynamic real-time collaborative task and found that around 200–300 ms after our actions are disturbed, our brain activity is modulated by the type of partner. Our results corroborate previous research on the neural basis of human-robot interactions. Furthermore, we show the feasibility of conducting research on collaboration between human and non-human partners with EEG. The results of our study suggest that non-human partners modulate how we perceive and evaluate joint actions. It is crucial that we found the differences between human and robotic partners during a dynamical coordination task, as it can have implications on the future of human-robot interactions and brain-computer interfaces. We speculate that our findings could improve already existing interfaces that use recognition of errors in real-time. It could be especially useful in situations when robots and humans have multiple interactions and it is important to distinguish between different partners. Further research into the origin of the observed differences might elucidate the neuronal substrate of understanding the behavior of a partner during joint action. Such research and application could further facilitate interactions between humans and robots in many environments.

## Data Availability Statement

Publicly available datasets were analyzed in this study. This data can be found here: https://osf.io/s6zbm/.

## Ethics Statement

The studies involving human participants were reviewed and approved by The Swedish Ethical Review Authority (Etikprövningsnämnden). The patients/participants provided their written informed consent to participate in this study.

## Author Contributions

PK, DK, and MB: conceived the study. AC, ALG, AK, and PK: designed the study. AG and MB: programmed the tablet and the robot. AC, ALG, AK, AG, and MT data collection. ALG and AK: major data analysis. AC, TK, BE, and PK: minor data analysis. AC, ALG, and AK: initial draft of the manuscript. AC, ALG, AK, AG, BE, MB, and PK: revision and finalizing the manuscript. All authors contributed to the article and approved the submitted version.

## Conflict of Interest

The authors declare that the research was conducted in the absence of any commercial or financial relationships that could be construed as a potential conflict of interest.

## Publisher's Note

All claims expressed in this article are solely those of the authors and do not necessarily represent those of their affiliated organizations, or those of the publisher, the editors and the reviewers. Any product that may be evaluated in this article, or claim that may be made by its manufacturer, is not guaranteed or endorsed by the publisher.
