# Supplementary material for: Coordinating With a Robot Partner Affects Neural Processing Related to Action Monitoring
Source: Front Neurorobot. 2021 Aug 11;15:686010. doi: 10.3389/fnbot.2021.686010 (PMC8386170; doi:10.3389/fnbot.2021.686010)
Supplement: Supplementary file 1 [file Image_1.PDF]

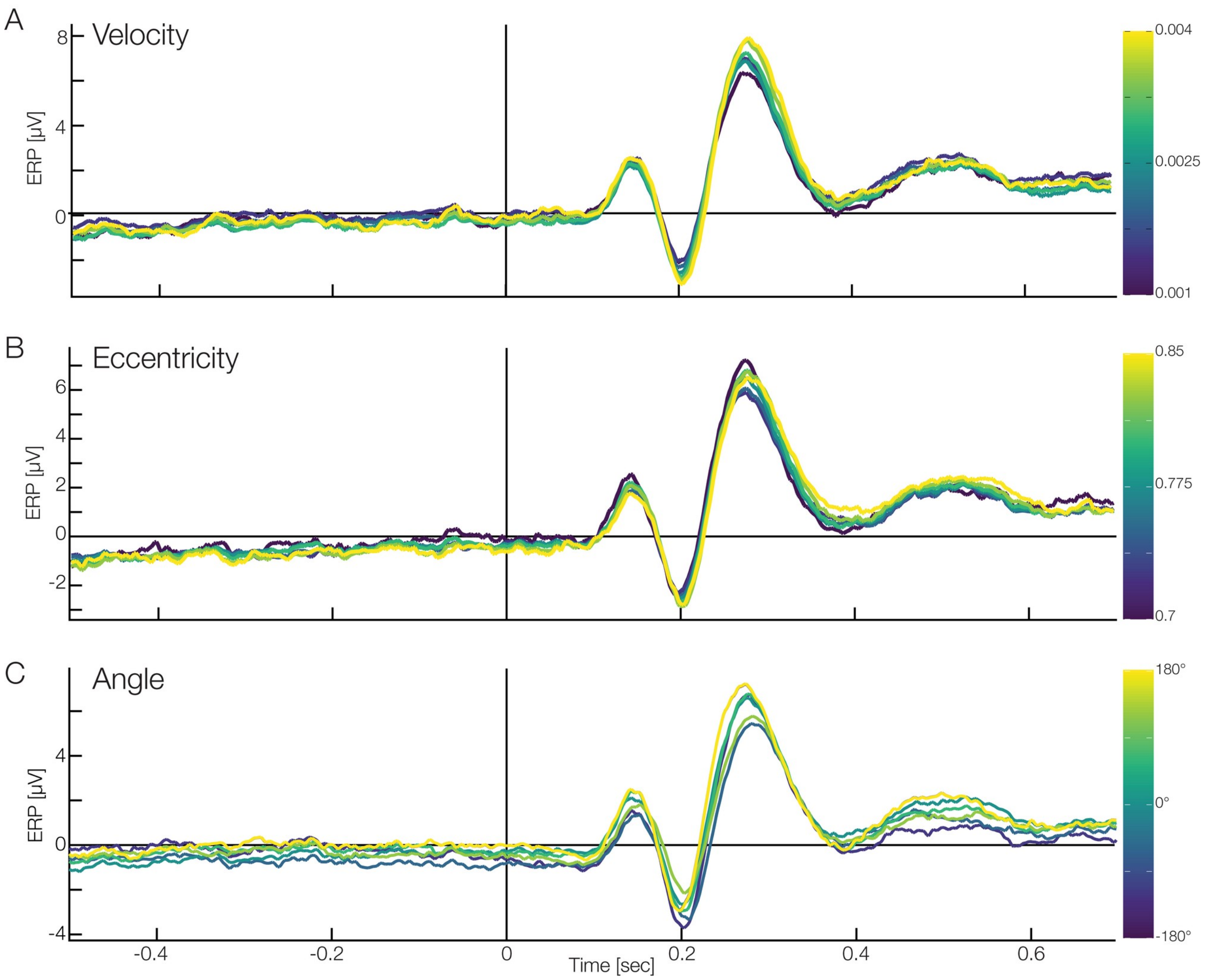

Figure: ERPs of the modeled covariates. (A) Velocity. The ERP shows a clear dependence on the speed of the ball with slower movement leading to a shallower activation. (B) Eccentricity. If the ball is positioned further outward, the ERP will show a stronger activation (C) Angle. We see that the opposing angles will also lead to opposing activity strength.
